# Emergence of kinky hair in Menkes disease

**DOI:** 10.1055/s-0044-1786761

**Published:** 2024-05-13

**Authors:** Hannah A. Oppenheim, Maria A. Montenegro

**Affiliations:** 1 University of California San Diego, Department of Neuroscience, San Diego California, United States.; 2 Rady Children's Hospital, San Diego California, United States.

A three-month-old male presented with developmental delay, rib fractures, and seizures. He had normal-looking hair. 1 A magnetic resonance imaging (MRI) scan of the brain was normal. Pathogenic *ATP7A* mutation was identified, which confirmed Menkes Disease. Only at 9 months of age did he develop patches of kinky hair.

**Figure 1:**
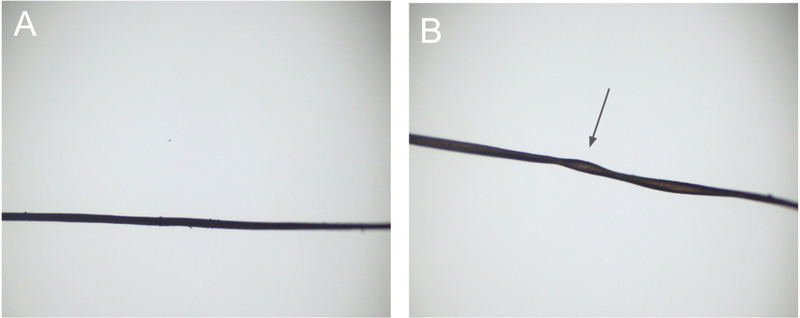
( **A** ) Normal hair at 3 months of age; and ( **B** ) hair strand at 9 months of age with classic *pili torti* seen in Menkes disease.

Parenteral copper histidine supplementation can modify Menkes disease progression if initiated within days after birth. 2 3 The clinical diagnosis relies on phenotypic presentation, especially sparse and lusterless scalp hair typically apparent by 2 to 3 months of age; however, our patient demonstrated that this feature may not appear until much later in life. Therefore, hair abnormalities should not be relied upon to initiate genetic testing ( Figure 1 ).
